# SIRT3: A New Regulator of Cardiovascular Diseases

**DOI:** 10.1155/2018/7293861

**Published:** 2018-02-13

**Authors:** Wei Sun, Caixia Liu, Qiuhui Chen, Ning Liu, Youyou Yan, Bin Liu

**Affiliations:** ^1^Department of Cardiology, The Second Hospital of Jilin University, 218 Ziqiang Road, Changchun 130041, China; ^2^Department of Neurology, The Liaoning Province People's Hospital, 33 Wenyi Road, Shenyang 110016, China; ^3^Department of Neurology, The Second Hospital of Jilin University, 218 Ziqiang Road, Changchun 130041, China

## Abstract

Cardiovascular diseases (CVDs) are the leading causes of death worldwide, and defects in mitochondrial function contribute largely to the occurrence of CVDs. Recent studies suggest that sirtuin 3 (SIRT3), the mitochondrial NAD^+^-dependent deacetylase, may regulate mitochondrial function and biosynthetic pathways such as glucose and fatty acid metabolism and the tricarboxylic acid (TCA) cycle, oxidative stress, and apoptosis by reversible protein lysine deacetylation. SIRT3 regulates glucose and lipid metabolism and maintains myocardial ATP levels, which protects the heart from metabolic disturbances. SIRT3 can also protect cardiomyocytes from oxidative stress-mediated cell damage and block the development of cardiac hypertrophy. Recent reports show that SIRT3 is involved in the protection of several heart diseases. This review discusses the progress in SIRT3-related research and the role of SIRT3 in the prevention and treatment of CVDs.

## 1. Introduction

Cardiovascular diseases (CVDs) are the leading causes of death worldwide, with >80% CVD-related deaths in low- and middle-income countries. Globally, the incidence of CVD-related deaths increased by 14.5% (95% confidence interval (95%CI): 12.1%–17.1%) between 2006 and 2016, although age-standardized death rates due to CVD decreased by 14.5% (95%CI: 12.5%–16.2%) over this time period [1]. By 2030, almost 23.6 million people are predicted to die from CVDs, mainly from heart disease and stroke [2, 3]. The molecular mechanisms of CVD include accumulation of reactive oxygen species (ROS), imbalance of vasoconstriction/vasodilation, chronic inflammation, and premature senescence, which are closely related to sirtuin-mediated regulation [4].

Sirtuins are a family of nicotinamide adenine dinucleotide- (NAD^+^-) dependent histone deacetylases, which are highly conserved across species from bacteria to humans [5–7]. It was first discovered in yeast as “silent mating type information regulator 2” (SIR2) in 1979 [8]. In mammals, seven SIR2 homologs (SIRT1–7) were identified, which differ in subcellular localization and mechanisms of biological regulation. SIRT3 is located in the mitochondrial matrix [9]; however, several studies suggest that it is also located in the nucleus and cytoplasm [10–12]. SIRT3 is a deacetylase that regulates the majority of mitochondrial lysine acetylation [13–15]. Recent studies show that SIRT3 plays important roles in cardiovascular physiology and pathology and describe the mechanisms via which SIRT3 regulates cardiac processes [16–25]. In this review, we will discuss current opinions on the role of SIRT3 in CVDs and speculate on its prospects for clinical application.

## 2. SIRT3: Function and Targets

### 2.1. SIRT3 and Energy Metabolism

More than 90% of ATP in the normal myocardium is derived from mitochondrial oxidative phosphorylation, followed by anaerobic glycolysis of glucose. Fatty acid beta oxidation is the main source of mitochondrial oxidative phosphorylation, followed by glucose, lactic acid, and ketone body aerobic oxidation [26]. SIRT3 regulates the enzymatic activity of the key enzymes of oxidative phosphorylation via deacetylation, thereby regulating mitochondrial energy metabolism (Table 1).

Reduced SIRT3 levels promote glycolysis via two mechanisms. First, the peptidylprolyl isomerase D (cyclophilin D) is in a highly acetylated state in the absence of SIRT3, which activates hexokinase II (HK2), a key glycolytic enzyme in the mitochondrial outer membrane. HK2 phosphorylates glucose to produce glucose-6-phosphate (G6P), the first step in most glucose metabolism pathways [27, 28]. Second, loss of Sirt3 increases ROS production, which stabilizes hypoxia-inducible factor- (HIF-) 1*α*, a transcription factor that regulates glycolytic gene expression [29, 30].

SIRT3-mediated deacetylation modifies and activates long-chain acyl-CoA dehydrogenase (LCAD), which is the key enzyme of fatty acid *β* oxidation, to promote fatty acid metabolism. Compared to wild-type mice, *Sirt3* knockout (*Sirt3*-KO) mice exhibit hallmarks of fatty acid oxidation disorders during fasting, including reduced ATP levels and intolerance to cold exposure [31]. In addition, SIRT3 can regulate other enzymes of fatty acid oxidation, such as medium chain-specific acyl-CoA dehydrogenase (ACADM) and acylglycerol kinase (AGK) [32]. Acyl-CoA synthetase short-chain family member 2 (ACSS2) plays a key role in lipogenesis by converting acetate to acetyl-CoA, which enters the tricarboxylic acid cycle (TCA) to promote oxidative phosphorylation and produce energy. Previous studies show that SIRT3 deacetylates and activates ACSS2 [33, 34]. In addition, SIRT3 regulates ketone body production by deacetylating and activating 3-hydroxy-3-methylglutaryl-CoA synthase 2 (HMGCS2), which is the rate-limiting enzyme in ketone-body biosynthesis [35, 36].

Most amino acids are decomposed by aminotransferase-mediated transfer of an *α*-amino moiety to *α*-ketoglutarate to form glutamate. SIRT3-mediated deacetylation activates glutamate dehydrogenase 1 (GLUD1), the enzyme that produces *α*-ketoglutarate from glutamate and is involved in both TCA cycle and ammonia metabolism [15]. SIRT3 can also upregulate the urea cycle (UC) through deacetylation and activation of ornithine transcarbamylase (OTC), the key enzyme of UC, suggesting that SIRT3 increases amino acid catabolism and ammonia detoxification during periods of metabolic stress [37]. Furthermore, SIRT3 regulates mitochondrial protein synthesis by deacetylation of the ribosomal protein MRPL10 [38].

The TCA cycle and electron transport chain (ETC) couple redox balance with ATP generation [39]. SIRT3-mediated deacetylation activates the components of the ETC complex, including NDUFA9 (complex I) [40] and SDHA (complex II) [41, 42]. SIRT3 also regulates ATP synthase activity [43]. Moreover, SIRT3 deacetylates IDH2, a key enzyme of TCA, which promotes oxidation of isocitrate to *α*-ketoglutarate and produces NADPH [44].

### 2.2. SIRT3 and Oxidative Stress

Oxidative stress causes extensive accumulation of intracellular ROS; destruction of proteins, lipids, and nucleic acids; membrane phospholipid peroxidation; and mitochondrial DNA mutations, which lead to severe cell damage and death. It is closely related to cardiac hypertrophy [17, 45–47], coronary atherosclerosis [48, 49], hyperlipidemia [37, 50], diabetes [51, 52], and other diseases. The mitochondrial ETC is the main source of ROS, and SIRT3 enhances the ability of the mitochondria to cope with ROS in multiple ways. The key superoxide scavenger, Mn superoxide dismutase (SOD2), can reduce superoxide production and protect against oxidative stress. SIRT3 directly regulates the activity of SOD2 by deacetylation [53–57]. Moreover, SIRT3 can sequester forkhead box O3a (FOXO3A) in the nucleus to increase the transcription of SOD2 and other key genes involved in antioxidation. Human serum FOXO3A and SIRT3 can be used as markers for ageing, with therapeutic potential for maintenance of healthy ageing [17, 58]. Second, SIRT3 deacetylates and activates the TCA cycle enzyme IDH2 and helps replenish the mitochondrial pool of NADPH [44]. NADPH is a key reducing factor that affects glutathione reductase, a part of the antioxidant defense system against cellular oxidative stress [44]. In addition, SIRT3 promotes effective electron transport via deacetylation of ETC complex components, which indirectly reduces ROS production [57].

### 2.3. SIRT3 and Apoptosis

Currently, the relationship between SIRT3 and apoptosis is not clear, and whether SIRT3 promotes or inhibits apoptosis is still controversial. However, SIRT3 was shown to mainly inhibit cardiomyocyte apoptosis in most studies. As mentioned previously, SIRT3 can inhibit apoptosis by regulating oxidative stress. In addition, SIRT3 also inhibits apoptosis by the following route: first, SIRT3 deacetylates and activates optic atrophy 1 (OPA1). Loss of OPA1 impairs mitochondrial fusion, perturbs cristae structure, and increases the susceptibility of cells toward apoptosis [59, 60]. Second, SIRT3 activates Ku70 by deacetylation. The activated Ku70 binds to Bax, which inhibits Bax-induced cardiomyocyte apoptosis [58]. Third, SIRT3 deacetylates cyclophilin D and closes the mitochondrial permeability transition pore (mPTP) to maintain the normal morphology of mitochondria, thereby inhibiting apoptosis [45, 61, 62].

## 3. SIRT3 in Cardiovascular Disease

### 3.1. SIRT3 in Ischemic Heart Disease

Ischemic heart disease (IHD) and cerebrovascular disease (stroke) together accounted for more than 85.1% of all CVD deaths in 2016. Total deaths from IHD increased by 19.0% (95%CI: 16.2%–22.1%), which contributed largely to the overall increase in total deaths from CVD in 2016 [1]. Severe ischemia can lead to a variety of pathological processes, including metabolic disorders and myocardial cell ultrastructure damage, which can be catastrophic for myocardial cells [63].

SIRT3 downregulation increases the susceptibility of cardiac-derived cells and adult hearts to ischemia-reperfusion (IR) injury and may contribute to a higher level of IR injury in the aged heart [22, 64]. *SIRT3* knockout leads to coronary microvascular dysfunction and impairs cardiac recovery post myocardial ischemia [18, 65]. Klishadi et al. reported that SIRT3 levels decreased after IR induced by left anterior descending artery occlusion [66]. The renin-angiotensin-aldosterone system (RAAS) is involved in ischemic injury [67, 68]. Angiotensin II downregulates SIRT3 [69], and drugs directly inhibiting RAAS normalized SIRT3 levels in the ischemic myocardium and improved cardiac function [40, 66, 70]. However, data on the instant effect of RAAS on SIRT3 during the acute ischemia is not available.

Atherosclerosis caused by vascular inflammation is also one of the pathophysiological mechanisms of IHD. Many studies have shown that SIRT3 is involved in the regulation of vascular inflammation. Trimethylamine-N-oxide (TMAO) activates nucleotide-binding oligomerization domain-like receptor family pyrin domain containing 3 (NLRP3) inflammasomes to induce vascular inflammation, which promotes atherosclerosis. TMAO was found to promote ROS generation, especially mitochondrial ROS, and inhibit SOD2 activation and SIRT3 expression in HUVECs and aortas from ApoE^−/−^ mice. Overexpression of SIRT3 resulted in protection against TMAO-induced activation of NLRP3 inflammasomes in endothelial cells. TMAO can also not further inhibit SOD2 and activate NLRP3 inflammasome in SIRT3 siRNA-treated HUVECs and aortas from SIRT3^−/−^ mice [71].

Moreover, a study of aerobic interval training showed that compared to the control group, *Sirt3* expression was reduced in the myocardium of rats with acute myocardial injury, while the interval of aerobic breathing resulted in increased *Sirt3* expression and protected against myocardial infarction-induced oxidative injury [72]. However, Winnik et al. observed that *SIRT3* knockout did not affect the progression of atherosclerotic lesions and plaque stability [73]. Therefore, the role of SIRT3 in atherosclerosis remains to be elucidated.

### 3.2. SIRT3 in Hypertrophy and Heart Failure

Hypertrophy is a compensatory response that results in cardiomyocyte death, fibrosis, and cardiac pressure or volume overload-induced heart failure because of structural changes in myocardial cells [74]. An increasing number of studies revealed that poor SIRT3 activity is one of the causes of cardiac hypertrophy and heart failure [17, 45, 75].

Four weeks after a transverse aortic constriction, the ejection fraction in *Sirt3*-KO mice was lesser than that in wild-type mice, which was accompanied by an increase in cardiac hypertrophy and fibrosis [19, 76]. *Sirt3*-KO mice showed a decreasing trend for palmitate oxidation, glucose oxidation, oxygen consumption, respiratory capacity, and ATP synthesis, whereas glycolytic rates were increased [19]. *Sirt3*-KO mice also showed abnormal lipid accumulation [77]. SIRT3 levels are reduced, and mitochondrial protein lysine acetylation is elevated in models of hypertensive heart failure, suggestive of impaired SIRT3 activity [78]. The low SIRT3 levels may be associated with the downregulation of PGC-1a [79]. Moreover, as a DNA repair enzyme, poly(ADP-ribose)-polymerase 1 (PARP-1) also uses NAD^+^ as a cosubstrate [80]. Overactivation of PARP-1 in failing hearts increases the competition between SIRT3 and NAD^+^, which leads to large-scale consumption of cellular NAD^+^ and a decrease in SIRT3 activity [80, 81]. In addition, RIP140, which is involved in the pathogenesis of cardiac hypertrophy and heart failure, also inhibits SIRT3 [82].

Recent studies show that SIRT3 regulates energy metabolism [30, 32], resists oxidative stress [17, 45, 47, 58], and prevents cardiac hypertrophy. Nicotinamide mononucleotide adenylyltransferase 3 (NMNAT3) is the only known enzyme of the NAD^+^ synthesis pathway that is localized in the mitochondrial matrix [83]. NMNAT3 is a SIRT3 substrate, deacetylation of which enhances its activity. Subsequently, NMNAT3 contributes to SIRT3-mediated antihypertrophic effects in cardiomyocytes by supplying NAD^+^ [84, 85]. Moreover, a study showed that SIRT3-LKB1-AMPK pathway activation improved cardiac hemodynamics and preserved the ejection fraction [25, 86]. SIRT3 can also protect against cardiac fibrosis by inhibiting myofibroblast transdifferentiation via the STAT3-NFATc2 [87] and *β*-catenin/PPAR-*γ* [88] signaling pathways.

In addition, emerging evidence indicates that impaired angiogenesis may contribute to hypertension-induced cardiac remodeling. A mouse myocardial fibrosis model induced by angiotensin II confirmed that SIRT3 enhanced mitochondrial autophagy, mediated by Pink/Parkin, and attenuated the production of mitochondrial ROS, restored vascular budding and tube formation, and improved myocardial fibrosis and cardiac function [89].

### 3.3. SIRT3 in Drug-Induced Cardiotoxicity

Drug-induced cardiotoxicity is now a common clinical practice. In particular, chemotherapeutic drug-induced cardiotoxicity has spawned the emergence of the interdisciplinary field of cardio-oncology. Recently, practice guidelines were published to guide clinical work in this interdisciplinary area [90].

Anthracycline chemotherapeutics such as doxorubicin (DOX) are commonly used for the clinical treatment of tumors. DOX induces cardiotoxicity via mitochondrial dysfunction [91]. A study showed that DOX reduced *SIRT3* expression and correspondingly increased mitochondrial protein acetylation, an effect that was more pronounced in *SIRT3*-KO mice [23, 92, 93]. *SIRT3* overexpression deacetylates and activates OPA1, which regulates mitochondrial dynamics and protects cardiomyocytes from doxorubicin-induced cardiotoxicity [23, 60, 93, 94]. Moreover, SIRT3 may also attenuate doxorubicin-induced cardiac hypertrophy and mitochondrial dysfunction via suppression of BNIP3 [95].

### 3.4. SIRT3 in Diabetic Cardiomyopathy and Cardiac Lipotoxicity

Diabetes and obesity are important risk factors for CVD. Studies show that the mitochondrial sirtuin family is involved in insulin resistance with diabetes mellitus [19, 20, 24].

SIRT3 can prevent and even reverse diabetes-induced retinal [96–98], skeletal [99–101], and cardiac damage [102, 103]. The SIRT3-FOXO3A-Parkin signaling pathway may play a vital role in the development of diabetic cardiomyopathy [102]. Melatonin protects against diabetic cardiomyopathy through MST1/SIRT3 signaling [104], and garlic protects patients with diabetic cardiomyopathy from oxidative stress by enhancing SIRT3 activity [105]. Moreover, *APLN* gene therapy increases angiogenesis and improves cardiac functional recovery in diabetic cardiomyopathy via upregulation of the SIRT3 pathway [103].

SIRT3 levels were low in the hearts of high-fat diet- (HFD-) fed obese mice, and cardiac lipotoxicity was high in *Sirt3* knockout mice [19, 20, 24]. SIRT3 possibly regulates lipotoxicity by promoting lipid metabolism, reducing fatty acid accumulation and oxidative stress injury [31, 32, 106], and restoring cardiac remodeling function [20, 24].

Oxidative stress is the common pathophysiology of diabetic cardiomyopathy and cardiac lipotoxicity [107, 108]. SIRT3 protects pancreatic beta cells from lipotoxicity by antagonizing oxidative stress-induced cell damage [109]. Thus, SIRT3 can inhibit diabetic cardiomyopathy and cardiac lipotoxicity.

### 3.5. SIRT3 in Hypertension

Hypertension, a common risk factor for diseases, such as cardiovascular, cerebrovascular, and kidney disease, has become a major risk factor for premature death and disability worldwide [110]. The roles of SIRT3 in hypertension are well documented. Waypa et al. first demonstrated that Sirt3 deletion does not augment hypoxia-induced ROS signaling or its consequences in the cytosol of pulmonary arterial smooth muscle cells, nor the development of pulmonary arterial hypertension (PAH) [111]. However, Paulin et al. more recently reported that SIRT3 is downregulated in a rat PAH model and in human PAH tissues. SIRT3^−/−^ and SIRT3^+/−^ mice develop PAH in a gene dose-dependent manner. Sirt3 gene therapy even reverses PAH in rats in vivo and human vascular cells in vitro [30]. Recently, Dikalova et al. showed that downexpression and redox inactivation of Sirt3 lead to SOD2 inactivation and contribute to the pathogenesis of hypertension [55]. These data suggest that Sirt3 responses may be cell type specific or restricted to certain genetic backgrounds.

## 4. Therapeutic Application

SIRT3 plays an important role in the development and progression of CVDs. Therefore, promotion of SIRT3 expression and activity via pharmacological pathways can delay the development of CVDs (Table 2).

### 4.1. Traditional Chinese Medicine

Components extracted from traditional Chinese medicine promoted SIRT3-mediated cardioprotective effects [112]. Resveratrol, which is abundant in grapes and blueberries, is the first compound that was shown to activate SIRT3 [113, 114]. A study demonstrated that resveratrol activates SIRT3 and downregulates the TGF-*β*/Smad3 pathway to improve myocardial fibrosis [115]. Polydatin, a monocrystalline and a polyphenolic drug extracted from the traditional Chinese herb *Polygonum cuspidatum*, protects cardiomyocytes against myocardial infarction injury by upregulating autophagy and improving mitochondrial biogenesis via SIRT3 activity [116]. Berberine decreases DOX-induced cardiotoxicity by activating SIRT3 [117]. Honokiol, a natural biphenolic compound derived from the bark of magnolia trees, blocks and reverses cardiac hypertrophy by activating SIRT3 [76, 118]. Pomegraniin A activates SOD2 through SIRT3-mediated deacetylation and reduces intracellular ROS [119]. Oroxylin A (OA), which is derived from the root of *Scutellaria baicalensis*, was found to be a SIRT3 activator in an in vitro model of cardiac myocyte insulin resistance [120] and plays roles in preventing myocardial contractile function and improving myocardial fibrosis and heart failure [121]. Curcumin, a phenolic compound extracted from the natural herb turmeric, can activate the PGC-1*α*/SIRT3 signaling pathway to protect against mitochondrial impairment, and it can also stimulate SIRT1 to have cardioprotective effects [122].

### 4.2. Small Molecule Activators of SIRT3

Inactivation of PIKFYVE, an evolutionarily conserved lipid kinase that regulates pleiotropic cellular functions, suppresses excessive mitochondrial ROS production and apoptosis through a SIRT3-dependent pathway in cardiomyoblasts [123]. Moreover, melatonin treatment alleviated cardiac dysfunction and ameliorated myocardial ischemia-reperfusion injury via SIRT3-dependent regulation of oxidative stress and apoptosis [79, 104, 124, 125]. Adjudin, which functions as an antispermatogenic agent, was found to attenuate oxidative stress and cellular senescence by activating SIRT3 [126]. Minocycline inhibited HIF-1*α*-mediated cellular responses and protected the blood-brain barrier and hypoxic brain injuries via activation of the SIRT3-PHD2 pathway. It implied that minocycline may have roles in IHD [127]. Mitochondrial intermediate peptidase (MIPEP), a mitochondrial signal peptidase (MtSPase), may promote the maintenance of mitochondrial quality during caloric restriction (CR), which extends lifespan and suppresses age-associated pathophysiology in various animal models by activating SIRT3 [128]. Metformin, a common drug for diabetes treatment, was found to modulate the appearance of atherosclerosis and reduce vascular events by increasing SIRT3 expression [129]. SIRT3 activity is dependent on the levels of cellular NAD^+^ and exogenous supplementation of NAD^+^ [78, 130–134]. Therefore, an increase in the levels of NMNAT3, the rate-limiting enzyme for mitochondrial NAD^+^ biosynthesis, also enhances the myocardium-protective role of SIRT3 [84]. Moreover, a variety of SIRT3-specific small molecules is being developed [135–137]. A recent study identified a novel SIRT3 activator, 7-hydroxy-3-(4′-methoxyphenyl) coumarin (C12), which binds to SIRT3 with high affinity, promotes deacetylation of MnSOD, and modulates mitochondrial protein acetylation and ROS [138].

### 4.3. Signaling Pathways

In addition, other signaling paths can also increase SIRT3 activity. EphB2 signaling-mediated *SIRT3* expression promotes MnSOD-mediated mtROS scavenging [139]. Mitochondrial cAMP/PKA signaling controls SIRT3 levels and proteolytic processing of OPA1 [59]. AMPK attenuates the cardiac remodeling parameters and improves cardiac function via the SIRT3/oxidative stress signaling pathway [140]. SIRT1 activation induces SIRT3 and the combined functions of the nuclear-mitochondrial triad switch glycolysis to fatty acid oxidation and immunity from activation to repression in acute inflammation [141]. A study that is expected to be used in clinical trials showed that PLGA-PNIPAM microspheres loaded with the gastrointestinal nutrient NaB specifically binds to SIRT3 and activates its deacetylase function, thereby inhibiting ROS generation and autophagy, promoting angiogenesis and protecting cardiomyocytes in acute myocardial infarction (AMI) [142]. The peroxisome proliferator-activated receptor gamma coactivator 1-alpha- (PGC-1*α*-) heme oxygenase- (HO-1-) SIRT3 pathway increases mitochondrial viability and provides metabolic protection [143].

### 4.4. MicroRNAs

MicroRNAs are single-stranded, noncoding small molecule RNAs that inhibit translation or induce target molecule degradation. Our previous study found that microRNA-210 can indirectly regulate the expression and activity of SIRT3 and promote cellular energy metabolism [144]. A study of *Drosophila* circadian rhythms found that microRNA-92a modulates neuronal excitability by suppressing the expression of SIRT2, which is homologous to human SIRT2 and SIRT3 [145]. Poulsen et al. found that high glucose levels coupled with oxidative stress resulted in the upregulation of microRNA28-5p, which directly inhibited expression of SIRT3 [146]. To date, the only microRNA for which SIRT3 was identified as a functionally relevant target was microRNA-421. MicroRNA-421 acts upstream of the SIRT3/FOXO3 pathway to modulate oxidant stress and lipid metabolism [147]. Although the study of the regulation of SIRT3 by microRNAs has only just begun, it may provide a new approach for the application of SIRT3.

## 5. Conclusion and Outlook

An important pathophysiological mechanism of cardiovascular disease is mitochondrial damage and dysfunction. As shown in Table 1, SIRT3 plays important roles in cellular energy metabolism, oxidative stress, and apoptosis. Moreover, it can potentially be used for protection against CVDs. Currently, small molecule drug screening for SIRT3 constitutes an important research direction. We believe that, in the near future, studies on SIRT3 will generate new approaches for the prevention and treatment of CVDs. SIRT3 can also regulate systemic inflammation, improving the whole body's metabolic balance. Although much progress has been made in the study of SIRT3, there are still many problems to be clarified: (1) the sirtuin family plays an important role in cardiovascular regulation; however, the physiological and pathological effects of SIRT4 and SIRT5 are unclear. (2) Sirtuins are the sentries of mitochondrial homeostasis; however, the interactomes of mitochondrial sirtuins are quite different from each other, which suggests that there is a diversity and complexity of sirtuin functions within the mitochondrion. A better understanding of the mechanism of mitochondrial regulation of CVD will be further advanced by studies of the regulatory networks acting among the sirtuin family members. (3) SIRT3, named “the longevity gene,” is closely related to the ageing of the human body. However, there is currently little data on the function of SIRT3 in ageing-related decline in cardiovascular functions. Further research to address these questions will be helpful in advancing our understanding of the roles of SIRT3 in CVD in order to facilitate the development of clinical diagnostic therapies.

## Figures and Tables

**Table 1:** Known targets of SIRT3 and function.

Function	Gene symbol	Gene name	References
*Energy metabolism*			
Glycolysis	PPID	Peptidylprolyl isomerase D (cyclophilin D)	[27]
Fatty acid oxidation	ACADL	Long-chain Acyl-CoA dehydrogenase (LCAD)	[31]
Ketone body synthesis	HMGCS2	3-Hydroxy-3-methylglutaryl-CoA synthase 2, mitochondrial	[35]
Acetate metabolism	ACSS2	Acyl-CoA synthetase short-chain family member 2	[33,34]
Urea cycle	OTC	Ornithine transcarbamylase	[37]
Amino acid catabolism	GLUD1	Glutamate dehydrogenase 1 (GDH)	[15]
Mitochondrial protein synthesis	MRPL10	Mitochondrial ribosomal protein L10	[38]
Oxidative phosphorylation	NDUFA9	NADH dehydrogenase (ubiquinone) 1 *α* subcomplex 9	[40]
	SDHA	Succinate dehydrogenase complex, subunit A, flavoprotein	[41,42]
	ATP5a	F_1_F_0_-ATPase subunit *α*	[43]
TCA cycle	IDH2	Isocitrate dehydrogenase 2, mitochondrial	[44]
*Oxidative stress*			
Transcriptional activation	FOXO3a	Forkhead box O3a	[17,58]
ROS	SOD2	Superoxide dismutase 2, mitochondrial (MnSOD)	[53–57]
*Apoptosis*	OPA1	Optic atrophy 1	[59,60]
	XRCC6	X-ray repair cross-complementing 6 (Ku70)	[58]

**Table 2:** Therapeutic application of SIRT3.

Categories	Representatives	Mechanism	References
Traditional Chinese medicine	Resveratrol	↑SIRT3 ↓TGF-*β*/Smad3	[113–115]
	Polydatin	↑SIRT3	[116]
	Berberine	↑SIRT3	[117]
	Honokiol	↑SIRT3	[76,118]
	Pomegraniin A	↑SIRT3/SOD2	[119]
	Oroxylin A	↑SIRT3	[120,121]
	Curcumin	↑PGC-1*α*/SIRT3	[122]

Small molecule activators of SIRT3	↓PIKFYVE	↑SIRT3	[123]
	Melatonin	↑SIRT3	[79, 104, 124, 125]
	Adjudin	↑SIRT3	[126]
	Minocycline	↑SIRT3/PHD2 ↓HIF-1*α*	[127]
	MIPEP	↑SIRT3	[128]
	Metformin	↑SIRT3	[129]
	NMNAT3	↑NAD^+^/SIRT3	[84]
	C12	↑SIRT3/MnSOD	[138]

Signaling pathways	EphB2 signaling	↑SIRT3/MnSOD	[139]
	cAMP/PKA signaling	↑SIRT3/OPA1	[59]
	AMPK	↑SIRT3	[140]
	SIRT1	↑SIRT3	[141]
	PLGA-PNIPAM-NaB	↑SIRT3	[142]
	PGC-1*α*-HO-1	↑SIRT3	[143]

MicroRNAs	MicroRNA-210	↓SIRT3	[144]
	MicroRNA-92a	↓SIRT2 (*Drosophila*, homologous to human SIRT2 and SIRT3)	[145]
	MicroRNA28-5p	↓SIRT3	[146]
	MicroRNA-421	↓SIRT3/FOXO3	[147]
